# Effect of High Hydrostatic Pressure and Pulsed Electric Fields Processes on Microbial Safety and Quality of Black/Red Raspberry Juice

**DOI:** 10.3390/foods11152342

**Published:** 2022-08-05

**Authors:** Génesis V. Buitimea-Cantúa, Iván Alejandro Rico-Alderete, Magdalena de Jesús Rostro-Alanís, Jorge Welti-Chanes, Zamantha J. Escobedo-Avellaneda, Mayra Cristina Soto-Caballero

**Affiliations:** 1 Escuela de Ingeniería y Ciencias, Tecnologico de Monterrey, Av. Eugenio Garza Sada 2501 Sur, Monterrey 64849, Nuevo León, Mexico; 2 Facultad de Ciencias Agrotecnologicas, Universidad Autonoma de Chihuahua, Av. Presa de la Amistad 2015, Cuauhtémoc 31510, Chihuahua, Mexico

**Keywords:** black/red raspberry juice, HHP, PEF, TPC, vitamin C, PME activity

## Abstract

Black and red raspberries are fruits with a high phenolic and vitamin C content but are highly susceptible to deterioration. The effect of high hydrostatic pressure (HHP 400–600 MPa/CUT-10 min) and pulsed electric fields (PEF, frequency 100–500 Hz, pulse number 100, electric field strength from 11.3 to 23.3 kV/cm, and specific energy from 19.7 to 168.4 kJ/L) processes on black/red raspberry juice was studied. The effect on the inactivation of microorganisms and pectin methylesterase (PME) activity, physicochemical parameters (pH, acidity, total soluble solids (°Brix), and water activity (*a_w_*)), vitamin C and phenolic compounds content were also determined. Results reveal that all HHP-treatments produced the highest (*p* < 0.05) *log*-reduction of molds (*log* 1.85 to 3.72), and yeast (*log* 3.19), in comparison with PEF-treatments. Increments in pH, acidity, and TSS values attributed to compounds’ decompartmentalization were found. PME activity was partially inactivated by HHP-treatment at 600 MPa/10 min (22% of inactivation) and PEF-treatment at 200 Hz/168.4 kJ/L (19% of inactivation). Increment in vitamin C and TPC was also observed. The highest increment in TPC (79% of increment) and vitamin C (77% of increment) was observed with PEF at 200 Hz/168.4 kJ/L. The putative effect of HHP and PEF on microbial safety, enzyme inactivation, and phytochemical retention is also discussed in detail. In conclusion, HHP and PEF improve phytochemical compounds’ content, microbial safety, and quality of black/red raspberry juice.

## 1. Introduction

Black raspberry (*Rubus occidentalis* L.) and red raspberry (*Rubus idaeus* L.) are two fruits, belonging to the Rosacea family, whose name is derived from the Anglo-Latin term raspise which means “a sweet rose-colored wine” [1]. These berries are in high demand by consumers due to their attractive color, flavor, and antioxidant content [2]. These characteristics are attributed to a wide range of biologically active substances including phenolics such as phenolic acids, flavonoids, anthocyanins, flavonols, and tannins, as well as vitamin C [3,4]. These compounds are responsible for antioxidant activity and various other health benefits, such as the prevention of inflammation disorders, cardiovascular diseases, or protective effects to lower the risk of various cancers [3]. Despite their high content in bioactive compounds, black and red raspberry fruits are susceptible to deterioration due to their high moisture content and smooth skin, making them vulnerable to physical damage and microbiological spoilage during harvest, packing, and storage, affecting their shelf-life which is limited to a few days (3–5 days), complicating their commercialization and distribution [5].

Traditional fruit processing methods have relied on high temperatures as a way to ensure prolonged shelf-life and food safety. However, thermal processes have some limitations causing severe damage to their sensory, nutritional, and functional properties [6]. Therefore, beverage processing is open to new technologies. Non-thermal technologies such as high hydrostatic pressure (HHP) and pulsed electric field (PEF) are growing increasingly in the juice industry. Both technologies can be used potentially to speed up the production process, improve the quality, develop new beverages or new features in conventional beverages, reach more stable beverages with better safety, and protect the sensory and nutritional quality of juice [7]. In this way, the non-thermal technologies have generated a lot of interest to process fruits or juices for their ability to increase extraction yields, and inactivate microorganisms, which enhance long-term microbial and physicochemical stability. Additionally, they allow the use of new fermentation biotechnologies with positive sensory impacts, as compared to conventional thermally processed foods. Besides, these technologies can be used as storage technology to keep stable beverage properties at room temperature. Thus, HHP and PEF are two attractive technologies to the food industry because more, flavorful, colorful, fresh, and nutrient-rich juice may be produced [8,9].

Recently, the applications of non-thermal technologies to promote phytochemical retention or improvement, and enzyme inactivation have been explored in fruit juice [8,10,11]. These effects have been attributed to HHP causing the modification of microfibrils in cell walls on plant cells, promoting the release of bioactive compounds from their intracellular compartments making them more bioaccessible [12]. While the PEF increases the permeability of membranes, they create larger openings (pores) through which water can be diffused to dehydrate and concentrate plant tissues, and also if these openings are reversible they provide for easier introduction of desirable components such as nutrients or flavor compounds [13,14].

Important advances have been made in the HHP and PEF applications to extend the shelf-life of food with minimal changes in food quality [15,16]. Several studies have reported the beneficial effect of these non-thermal technologies on the stabilization and quality of strawberry juice and pure [17,18,19,20,21,22,23]. In the fruit juice industry, pectin methylesterase (PME) is an enzyme of great concern [24]. This is because it modifies the physical appearance of juices [8] And affects the viscosity and stability of the suspended particles in the juice cloud [25,26]. This is attributed to the de-esterification of pectin molecules and the subsequent formation of calcium bridges between free carboxyl groups of adjacent pectin molecules, which contributes to the loss of cloudiness in juice [8].

Considering that HHP and PEF technologies could be an attractive alternative to preserve the functional and sensorial properties of fruit juice, there is no information about the effect of HHP and PEF on the stabilization of black and red raspberry. This work aimed to determine the effect of HHP and PEF on black and red raspberry juice stabilization, evaluating the inactivation of the microbial organism (molds, and yeasts), and PME activity, as well as on the phytochemical parameters and vitamin C and phenolic compounds content.

## 2. Materials and Methods

### 2.1. Chemicals

Acetic acid (99.9%), *L*-ascorbic acid (AA), methanol, citrus pectin (galacturonic acid ≥74.0 %), ammonium acetate, gallic acid (GA), and Folin–Ciocalteu’s phenol reagent were purchased from Sigma-Aldrich (St. Louis, MO, USA). Sodium carbonate, sodium chloride, and sodium phosphate dibasic were purchased from J.T. Baker, (Ecatepec de Morelos, Mexico). Potassium chloride and sodium hydroxide were purchased from Fermont (Mexico City, Mexico). Water was provided by a Milli-Q water purification system (Millipore, Germany).

### 2.2. Plant Material and Juice Preparation

Black raspberry (*Rubus ulmifolius* L.) and red raspberry (*Rubus idaeus* L.) fruits from Organic Driscoll’s, México, in the mature stage for consumption were purchased in a local supermarket in Monterrey, Mexico. The fruits were carefully selected to avoid deteriorated berries and they were then washed with tap water. Then, 3.3 kg of each berry (1:1 *w*/*w*) was mixed using an industrial blender (JR-Torrey, Mexico) for 1 min until obtaining a homogenous sample. Concentrated juice was diluted at 70% by adding distilled water. The final juice has 7.5 °Brix and the pH was adjusted to 3.25 with distilled water and citric acid, respectively [10]. After that, the juice was immediately subject to HHP and PEF treatments.

### 2.3. Physicochemical Analysis

The pH of the black raspberry/red raspberry juice was determined by the official method (AOAC 981.12.) using a potentiometer (Thermo Fisher Scientific, Orion 3-star, USA), and the TSS was expressed as °Brix using a refractometer (ATAGO, Model PAL-α), water activity (*a_w_*) was determined with an AquaLab (series 3, Decagon Devices, Inc., Pullman, WA, USA) at room temperature (25 °C) [9], and the titratable acidity (%TA) was determined by the burette method, against NaOH (0.1 N) solution by using phenolphthalein as the indicator (AOAC, 1990).

### 2.4. HHP Treatments

For HHP treatments, 30 g of juice was placed in polyethylene bags (Filmpack S.A. de C.V., Guadalupe, Nuevo León, Mexico) and vacuum-sealed (Torrey, México) for HHP treatments in an AVURE 2 L HHP unit (Columbus, OH, USA) using water as a pressurizing medium at room temperature (25 °C) as an initial condition (20 °C). Samples were treated at 400, 500, and 600 MPa during the come-up time (CUT), 2, 5, and 10 min. Each treatment was carried out by duplicate and untreated samples were used as a control. Due to adiabatic heating during the holding time, the average process temperatures were 27, 29, and 31 °C at 400, 500, and 600 MPa, respectively [10]. Immediately after processing, the juice was analyzed for microbial and enzyme inactivation, and phytochemical content.

### 2.5. PEF Treatments

The juice was treated in a continuous-flow, system ELCRACK-HVP 5 (DIL, Quakenbrueck, Germany). The PEF system delivers square-wave pulses in bipolar mode and is composed of two cells that have two stainless steel electrodes separated by a gap of 7 mm at a flow rate of 60 L/h. The juice samples were treated at frequencies of 100, 200, and 500 Hz, with a pulse width of 25 μs, a pulse number of 100, an electric field strength from 11.3 to 23.3 kV/cm, and specific energy from 19.7 to 168.4 kJ/L. About 100 mL of the sample was collected at the outlet of the equipment, placed in polyethylene bags, and stored at 4 °C until analysis. Each treatment was carried out by duplicate and untreated samples were used as a control. Immediately after processing, the juice was analyzed for microbial and enzyme inactivation, and phytochemical content.

### 2.6. Microbiological Evaluation

Molds and the yeast population of the black/red raspberry juice were determined in the untreated juice and the treated juice immediately after processing by plate count agar according to Official Methods of Analysis [27,28]. After HHP and PEF treatments, 10 mL of the sample was homogenized with 90 mL of 0.1% sterile peptone water. Similarly, serial decimal dilutions of the treated samples and the control were made. Samples were seeded on Potato Dextrose Agar (PDA) and incubated at 28 °C for 5 days. The reduction of the viable cell was expressed as the decimal logarithm of the microbial enumeration [29].

### 2.7. Pectin Methylesterase (PME) 

PME activity was measured according to the methodology indicated by Escobedo-Avellaneda et al. [10]. Briefly, 20 g of beverage (W_sample_) was mixed with 20 mL of a solution consisting of 1% citrus pectin and 0.1 M NaCl previously maintained at 44 °C on a hot plate (CTR scientific, Mexico City, Mexico) to have a final temperature of 30 °C after mixing with the juice. The mixture was maintained at constant agitation (1200 rpm) at 30 °C (CTR scientific, Mexico City, Mexico) and then the pH (AOAC 981.12., (Orion 3 star, Thermo Fisher Scientific Inc., Waltham, MA, USA)) was immediately adjusted to approximately 7.0 with a 2N NaOH solution and readjusted at pH 7.7 with a 0.5 N NaOH solution. An excess of 30 µL (V_NaOH_) of 0.5 N NaOH (N_NaOH_) was added, and the time (t) required to return to pH 7.7 was registered. The PME activity (At) at each treatment condition was calculated with Equation (1) and the PME residual activity (RAt) was expressed as a percentage of the untreated sample with Equation (2).

At = (N_NaOH_ × V_NaOH_) Wt
(1)

RAt = PME _treated sample_ × 100 PME _untreated sample_ (2)

### 2.8. Vitamin C

The vitamin C content was determined according to that described by Escobedo-Avellaneda et al. [30] with some modifications. Vitamin C in the samples was extracted with TCA 6% using a 1:4 sample-solvent ratio and the mixture was centrifuged at 12,500 g/4 °C for 5 min using a Galaxy 16DH centrifuge (VWR International LLC, Radnor, PA, USA). The supernatant (50 µL) was mixed with 25 µL of potassium phosphate buffer 75 mM, pH 7. For the analysis of reduced ascorbate, 50 µL of distilled water was added to the mixture, then it was incubated for 10 min at room temperature (25 °C). Then 335 µL of a mixture of TCA 10%: H_3_PO_4_ 43%: bipyridyl 4%: FeCl_3_ 3%; in a percentage content of 33%, 26.7%, 26.7%, and 13.3%, respectively were added to all assay tubes. After 1 h incubation at 37 °C, 200 μL of the sample was transferred to a Costar clear 96-flat bottom well microplate (Thermo Fisher Scientific Inc., Waltham, MA, USA), and the absorbance was measured at 525 nm (maximum absorption point confirmed by wavelength scan 400 to 650 nm) in a microplate reader (Synergy HT, BioTek Instruments, Inc., Bad Friedrichshall, Germany). A five-point calibration curve (R^2^ = 0.9948) in a 0.1–1.0 mM range of AA was used to obtain vitamin C concentrations as mg AA/100 g sample on a wet basis. Then, three replicates with triplicates analyses were performed (*n* = 9) [30].

### 2.9. Total Phenolic Compounds (TPC)

Total phenolic compounds were measured according to the method described by Escobedo-Avellaneda et al. [30]. Phenolic extracts were prepared using 25 mg of the juice mixed with 2.5 mL of methanol: water (1:1 *v*/*v*). The mixture was vortexed at 3000 rpm for 1 min (model 945303, VWR International LLC, USA) and placed in a water bath at 90 °C for 2 h (Thermo Fisher Scientific Inc., Waltham, MA, USA) shaking in a vortex every 30 min. After that, the sample was centrifuged at 5000 g/4 °C for 10 min. The supernatant was recovered and made up to 5 mL with methanol. For the phenolic analysis, 50 µL of the methanol extract was mixed with 650 µL of distilled water and 50 µL of the Folin–Ciocalteau reagent. After 5 min at room temperature (25 °C), 250 µL sodium carbonate 0.5 M was added. After 2 h incubation at 37 °C, 200 μL sample was transferred to a Costar clear 96-flat bottom well microplate (Thermo Fisher Scientific Inc., Waltham, MA, USA), and the absorbance was measured at 765 nm in a microplate reader (Synergy HT, BioTek Instruments, Inc., Bad Friedrichshall, Germany). A six-point calibration curve (R^2^ = 0.9966) in a 50–300 ppm range of GA was used to obtain phenolic concentrations as mg GAE/100 g sample on a wet basis. Following this, three replicates with triplicates analyses were performed (*n* = 9) [30].

### 2.10. Statistical Analyses

Results were reported as the mean and standard deviation of three independent samples (triplicate). The analyses of variance (ANOVA) were performed at each treatment and a Tukey test at a 95% CI was carried out to identify significant differences between mean values (Minitab^®^ 16.1.1 Statistical Software for Windows, USA).

## 3. Results

### 3.1. Inactivation of Molds and Yeast by HHP 

High-acid food products like fruit juice are prone to mold and yeast spore-formers contamination. Therefore, in this work, the effect of the HHP and PEF-treatments on the inactivation of molds and yeasts in black/red raspberry juice was evaluated (Table 1).

Results showed that the initial microbial population of molds and yeasts was 5.30 × 10^3^ and 1.55 × 10^3^ CFU/mL, respectively. Significant differences were observed in inactivation levels depending on the applied pressure and processing time. The HHP treatments at 500 and 600 MPa achieved a 3.72-*log* reduction of molds inhibition during all conditions tested (CUT, 2, 5, and 10 min). The application of 400 MPa under CUT condition led to less than 1.85-*log* reduction, while, at the same pressure (400 MPa) but with an increment in time of 2 min achieved a 3.72-*log* reduction of molds inhibition. This suggests that HHP-treatment of 400 MPa required at least 2 min to produce a microbial inactivation, while higher pressures such as 500–600 MPa require at least CUT conditions to produce 3.72-*log* reduction.

Regarding the effect of the HHP-treatments on yeast inactivation, it was observed that it was completely inactivated (3.19-*log* reduction) at all conditions tested. Similar inactivation results have been reported in different juice drinks [31]. Confirming that HHP technology is suitable for microbial inactivation on black/red raspberry juice. The HHP results suggest that a differential effect on molds and yeast inactivation by pressure was obtained and the yeast was the most affected. Cheftel [32] also observed that yeast is most sensitive to pressure rather than mold. However, both yeast and molds are eukaryotes organisms with cell nuclei and membrane-bound organelles in the kingdom of Fungi. The main difference is that yeast is a unicellular type of fungi whereas mold is multicellular filaments of the fungi. The mechanism by which HHP-induced inactivation of microorganisms has not yet been elucidated. However, it has been reported that HHP cause morphological, biochemical, and genetic alterations in microorganism cells [32]. Therefore, some of these effects could be involved in the inactivation of yeasts and molds in the black/red raspberry juice obtained in this work.

### 3.2. Inactivation of Molds and Yeast by PEF 

The initial microbial population of molds (5.30 × 10^3^ UFC/mL) and yeasts (1.55 × 10^3^ UFC/mL) during the PEF treatments was less reduced in comparison with HHP treatments (Table 1). With PEF technology a mold’s inactivation ranging from 0.19-*log* to 2.12-*log* reduction was observed in the treatments, indicating that did not achieve total molds’ inactivation under any of the PEF-conditions tested, while a yeast population of 1.25 × 10^3^ to 6.50 × 10^1^ UFC/mL was obtained in PEF treatments with the frequency of 100 Hz and specific energy of 19.7 and 79.7 kJ/L, respectively. In addition, at a higher frequency such as 200 or 500 Hz and specific energy from 42.9 to168.4 kJ/L, a complete yeast inactivation was obtained. According to these microbial inactivation results, the yeast inactivation in black/red raspberry juice requires at least a frequency of 200 Hz and 42.9 kJ/L. Li et al. [33], reported similar results, they observed a three-cycle *log* reduction in the yeast population treated with the frequency of 200 Hz. Overall results, it was observed that yeast is more sensitive to PEF treatment rather than molds.

PEF inactivation is attributed primarily to the electroporation of the cell membrane. The creation of transient membrane pores during electroporation increases membrane conductivity resulting in a structural rearrangement of the membrane phospholipids [34]. The rapid change in membrane conductance results in an elevation in the intracellular field, which may produce biochemical changes inside the cells [35]. This then leads to microbial inactivation. Thus, the PEF treatment controls the number and size of pores produced and the inactivation grade. In yeast, the high lethal injury is caused by PEF at the frequency of 200 Hz., while in molds, the PEF treatments tested did not produce a lethal injury. This differential inactivation can be attributed to structural composition differences between molds and yeast. Considering that PEF affects the membrane in microorganisms is important to note that the fungal cell wall is located outside the plasma membrane and is the cell compartment that mediates all the relationships of the cell with the environment [36]. Therefore, the cell wall is the main barrier to membrane electroporation. In the molds, the cell wall is composed of chitin, glucans, and *N*- and *O*-linked oligosaccharides, while in yeast it is composed of chitin, β-glucans, and mannans [33]. Therefore, these differential compositions would be contributing to the higher sensitive effect of PEF by yeast rather than molds.

Overall, the combination of the microorganism’s characteristics with PEF-treatments provides promising methods for juice pasteurization, prolonging its shelf life and retaining the juice’s physicochemical properties. However, further studies should be conducted to examine the mechanism underlying the effect of PEF on yeast and mold inactivation.

### 3.3. Physicochemical Parameters of the Black/Red Raspberry Juice

The results of the pH, titratable acidity (% citric acid), total soluble solids (°Brix), and water activity (*a_w_)* values of the black/red raspberry juice subject to HHP and PEF treatments are shown in Table 2. 

#### 3.3.1. pH Value

The HHP and PEF control of the black/red raspberry juice presented a pH value of 3.26 ± 0.01 and 3.31 ± 0.01, respectively. These values presented an increment statistically significative (*p* < 0.05) after the treatments of HHP and PEF. In the HHP-treated juice, the pH value was in the range of 3.39 to 3.45, while in the PEF-treated juice the pH value was from 3.36 to 3.38. Suggesting that HHP-treatments produce a higher effect on the pH value in comparison with PEF-treatments (Table 2).

In the black/red raspberry juice treated with HHP, the highest pH value was observed in the HHP-treatment of 500 MPa/10 min (3.45 ± 0.01), followed by 400 MPa/10 min (3.43 ± 0.01) and 600 MPa/10 min (3.41 ± 0.01). The lowest pH value was observed in the HHP-treatment of 400 MPa/2 min (3.36 ± 0.01). These results suggest that the change in pH value was dependent on the pressure and time of the treatment. At the pressure of 400 and 500 MPa, the pH increases with the increment in the treatment time, thus, a higher pH value is observed during the treatment time of 10 min rather than 2 min; while at the pressure of 600 MPa the contrary case is observed, the higher pH value is observed in the CUT, rather than 10 min of treatment. For the PEF-treated juices, the highest (*p* < 0.05) pH value (3.38 ± 0.01) was obtained in the PEF-treatments of 100 Hz/19.7 kJ/L, 500 Hz/113.8 kJ/L, and 100 Hz/79.7 kJ/L, while the lowest pH value (3.36 ± 0.01) was in the treatment of 200 Hz/42.9 kJ/L. This suggests that frequency plays an important role in the change in pH value, i.e., the higher increment in the pH value was observed at the highest frequency (500 Hz) and the lowest frequency (100 Hz); while the lowest increment in pH value was observed at the frequency of 200 Hz. The pH value of the HHP and PEF-treated black/red raspberry juice and untreated juices classifies them as acidic products, as reported by Aganovic et al. [37]. The acidic pH value in berries, including black and red raspberries, is attributed to the presence of organic acids (such as citric acid, malic acid, tartaric, oxalic, and fumaric acid), vitamin C, and specialized metabolites, such as polyphenols, which are shown to have health-promoting properties [38].

#### 3.3.2. % Acidity

Titratable acidity (TA) measures the total acid concentration and salts contained in a fruit [39]. In this work, the TA of the juice was expressed as a percentage of citric acid. The juice controls presented a TA of 1.0% of citric acid, while a lower value was statistically significant (*p* < 0.05) for all HHP (0.8%) and PEF (0.9%) treated juice obtained (Table 2). These results reveal that HHP and PEF treatments increase the acidity in the juice. This effect would be attributed to the gradual migration of organic acids from the intracellular matrix of cells, being ruptured by HPP or PEF treatments. Thus, the increment in %TA is related to the release of the citric acid, malic acid, tartaric, oxalic, and fumaric acid), vitamin C, and specialized metabolites, such as polyphenols, the main organic acids present in berries which are shown to have health-promoting properties [38]. An increment in the %TA has also been attributed to the increase in the concentration of powerless ionized acid and their salts. As well as acid formation, reducing sugar oxidation, and polysaccharide degradation or the breakdown of uronic acid and pectin substances [40,41]. Therefore, according to the aforementioned, once the migration of organic acids occurs, they probably are partially ionized increasing the powerless ionized, producing an increment in %TA.

#### 3.3.3. TSS (°Brix)

In fruits, sweetness (fructose, glucose, and sugar content) is one of the desirable characteristics of commercially grown and a lack of it has been associated with low total soluble solids (TSS, expressed as °Brix) content and pH value [42,43]. In this work, the TSS in the black/red raspberries juice control was 7.5 ± 0.06 °Brix, while in the HHP and PEF-treated juice a statistically significant increment (*p* < 0.05) was observed. All HHP-treated samples presented 8.0 ± 0.06 °Brix and all the PEF-treated juices showed 8.5 ± 0.06 °Brix. This increment was attributed to the release of compounds from the cell which dissolve in the aqueous phase and become part of the fruit juice, incrementing the TSS [44]. Just as well, the enzymatic processes also lead to increased sugar contents [45]. Thus, the HHP and PEF treatments in juice could also produce an increment in the activity of the enzyme responsible to produce sucrose and other sugars resulting from starch reserves and inter-conversion of the released sugars [45].

#### 3.3.4. Water Activity (*a_w_*)

Water activity (*a_w_*) measures the availability of free water in a food system that is responsible for any biochemical reactions [46]. Thus, the *a_w_* can determine a food’s shelf stability [47]. The *a_w_* value in the controls and the HHP and PEF-treated juice of black/red raspberries were in the range of 0.97–0.98, and these values were statistically equal (*p* > 0.05), indicating that HHP and PEF treatment do not produce an effect in *a_w_* value. Generally, foods with an *a_w_* value < 0.6 are microbiologically more stable [46]. Therefore, the high *a_w_* value in the juice could be favorable to microbial growth, and any biochemical reactions responsible for juice deterioration.

Considering all of the physicochemical parameter results of the black/red raspberries juice, it was observed that HHP and PEF treatments produce an increment in the pH value, %TA, and °Brix, while the *a_w_* value was not affected (*p* > 0.05). 

### 3.4. PME Activity

**Figure 1:**
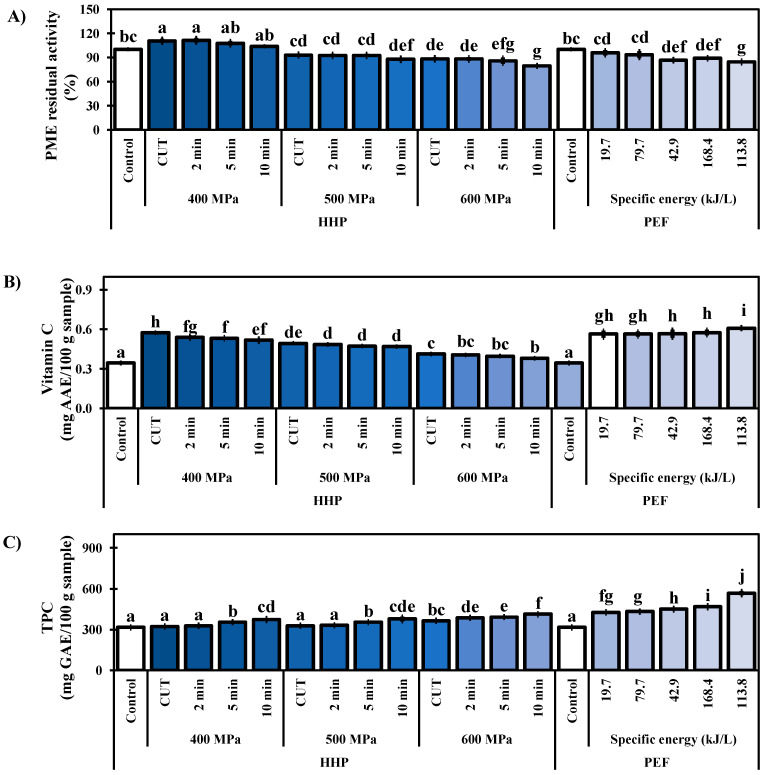
Effect of the HHP and PEF treatments to pasteurize black/red raspberry juice. (**A**) Inactivation of PME, (**B**) Vitamin C, and (**C**) TPC. Mean value ± standard deviations (*n* = 9). The values with different superscript letters are statistically different (*p* < 0.05).

In this work, the residual PME activity of the black/red raspberry juice untreated (controls) and treated under different conditions of HHP and PEF treatments was determined (Figure 1A). Treatments at 400 MPa generated increment in PME activity (*p* < 0.05) from 6 (400 MPa/10 min) to 14% (400 MPa/CUT). In contrast, the treatment at 500 and 600 MPa caused a reduction from 7 to 22% in the PME activity (*p* < 0.05). The treatment at 500 MPa caused a reduction in PME activity (*p* < 0.05) at times of 10 min (13%), 5 min (9%), 2 min (7%), and CUT (7%). For samples subjected to 600 MPa, the same behavior was observed; however, a higher PME activity reduction in comparison with 500 MPa was observed. In the HHP at 600 MPa, the higher reduction (*p* < 0.05) was obtained after 10 min (22%), followed by 5 min (17%), 2 min (12%), and CUT (12%). These results reveal that among the HHP-treatments, the highest inhibition (*p* < 0.05) was observed with the HHP-treatment of 600 MPa/10 min (22% of reduction). Regarding PEF treatments, the higher inhibition was observed at 200 Hz/168.4 kJ/L (19%), followed by 100 Hz/79.7 kJ/L (13%), and 500 Hz/113.8 kJ/L (13%); while the lowest inhibition was obtained at 100 Hz/19.7 kJ/L (7%) and 200 Hz/42.9 kJ/L 9%). Overall results, the HHP and PEF treatments decrease the PME activity. This reduction was higher with the HHP-treatment at 600 MPa/10 min (22%), followed by PEF at 200 Hz/168.4 kJ/L (19%).

According to the PME activity, for HHP-treatments two effects were observed, an increment and a reduction of the PME activity, while for PEF only decrements in the PME activity were observed. The increment of the PME activity during the HHP treatment at 400 MPa (CUT, 2, 5, and 10 min) would be related to the enzyme liberation of the structure cell after the HHP-treatment. Once the enzyme and its substrate are decompartmentalized, they interact, and the enzyme reaction takes place leading to increased enzyme activity in the juice treated. This increment is also attributed to the reversible configuration of the enzyme after HHP-treatment, leading to structural changes that favor enzyme-substrate interaction [48] or the release of a second active center [49]. Thus, the pressure of 400 MPa was favorable to the interaction of the PME with the highly methylated pectin, leading to demethylation [50]. In addition, the baro-resistance of the enzymes is also favorable to the increment of enzyme activity. In fruits, it has been observed that baro-resistance of the enzyme is attributed to the sugar content, which causes an interference with enzyme stability and re-folding during treatment [51]. In addition to the ascorbic acid content in fruit, which acts as an enzyme protective agent during HHP-treatment [52]. On other hand, the reduction of the enzyme activity by the HPP treatments at 500 and 600 MPa and PEF treatments at 200 Hz/168.4 kJ/L, 100 Hz/79.7 kJ/L, and 500 Hz/113.8 kJ/L are attributed to the irreversible protein denaturation, which modifies functionality resulting in enzyme catalytic activity reduction. This protein denaturation was dependent on the HHP and PEF treatment conditions applied, i.e., the highest pressure tested (600 MPa and the PEF treatment 200 Hz/168.4 kJ/L) produced a higher enzyme activity reduction, while the lower treatment (500 MPa/CUT and 100 Hz/19.7 kJ/L) produced a lower enzyme activity reduction.

### 3.5. Vitamin C Content

Berries are fruits with high content of vitamin C (*L*-ascorbic acid), therefore, the effect of the HHP and PEF treatments on the vitamin C in the black/red raspberry juice was determined (Figure 1B). In the control, the vitamin C content was 0.344 mg AAE/100 g of the sample, and in the HHP-treatments a concentration in the range of 0.380–0.574 mg AAE/100 g of the sample was observed, which indicates an increase of 11–67% (*p* < 0.05) in comparison with the control. The PEF treatments presented a concentration of 0.564–0.609 mg AAE/100 g of the sample, indicating an increase of 64–77%. In the HHP-treatments of 400 MPa, the CUT caused an increment of 67%, and in the treatment time of 2, 5, and 10 min, the increment was 56, 55, and 50%, respectively. The HHP-treatments of 500 and 600 MPa present the same behavior, the higher increment was in the CUT, followed by 2, 5, and 10 min. However, 500 and 600 MPa presented a lower increment (*p* < 0.05) in comparison with the treatment of 400 MPa. In the PEF treatments, vitamin C content also presents an increment (*p* < 0.05). In the treatment of 100 Hz/19.7 kJ/L, 200 Hz/42.9 kJ/L, and 500 Hz//113.8 kJ/L an increment of 64 and 65%, was obtained, while in the treatments of 100 Hz/79.7 kJ/L and 200 Hz/168.4 kJ/L, increments of 67 and 77%, respectively, were observed. The higher vitamin C content observed could be due to the capacity of HHP and PEF treatments to release the compound from compartments containing high concentrations of vitamin C, such as the cytosol and vacuole, producing an increment in the content [18,53,54].

### 3.6. Total Phenolic Content

The phenolic compounds play a fundamental role in the nutraceutical composition of black/red raspberry juice. In this study, the effect of HHP and PEF treatments on the total phenolic compounds (TPC) in the juice was determined (Figure 1C). The TPC in the control juice was 316.3 mg GAE/100 g of the sample. Some HHP conditions caused increments of 2 to 30% in the TPC of juice, in which the TPC values ranged from 321.9 to 412.2 mg GAE/100 g of the sample, the highest increment was obtained at 600 MPa/10 min. The TPC also increased in the juice treated by PEF. The TPC content in juice processed by PEF varied from 426.4 to 567.2 mg GAE/100 g of the sample, representing increments from 35 to 74% as the field intensity and frequency increased. The increments in TPC would be related to the phenolic release of the vacuole from the vegetable cell. Especially, the PEF treatments produce a greater effect on the release of this metabolite from cellular compartments compared to HHP treatments. This suggests that HHP and PEF produce chemical modifications of the food matrix, leading the compounds into more bioaccessible and bioavailable forms [55,56]. The chemical modifications include the cleavage of covalent or hydrogen bonds that attach phenolic compounds to matrix macromolecules or the damaging microstructural barriers such as cell walls that impede the release from the matrix [55]. In this way, the HHP and PEF treatment enhance phenolic compounds’ bio-accessibility and bioavailability in the black/red raspberry juice. 

The increase in the phenolic compounds’ content by the HHP and PEF treatments can improve the intensity of color in the final beverage, as well as the nutritional value and antioxidant activity, attributed to the phenolic compounds’ bio-accessibility and bioavailability. In berries, the main phenolic compounds include flavonoids, anthocyanins (i.e., cyanidin glucosides and pelargonidin glucosides), flavonols (quercetin, kaempferol, myricetin), flavanols (catechins and epicatechin). As well as phenolic acids (hydroxybenzoic acids and hydroxycinnamic acids) and hydrolyzable tannins, such as ellagitannins. Therefore, the increment in TPC would be related to the increment of these components, which are mainly responsible for the health benefits of the consumption of berries.

## 4. Discussion

### Putative Effect of the HHP and PEF on Black/Red Raspberry Juice

**Figure 2:**
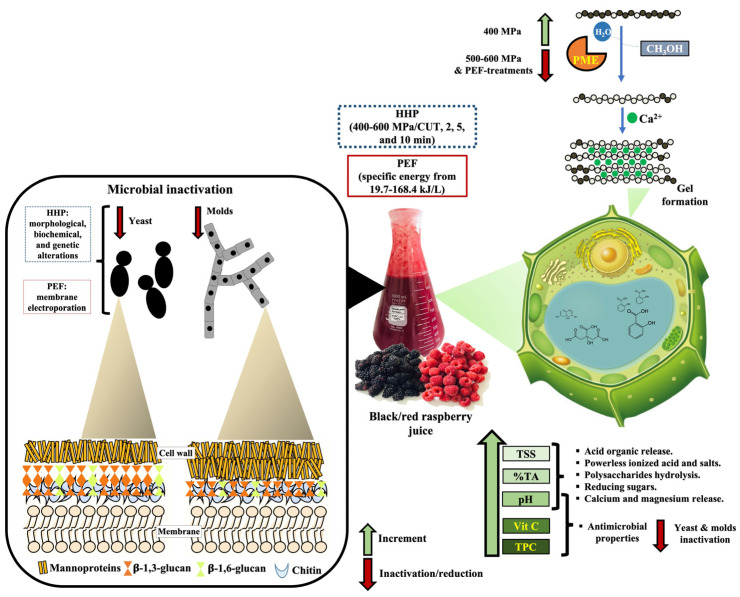
Putative effect of the high hydrostatic pressure and pulsed electric field on the microbial safety, enzyme inactivation, and phytochemical retention in the black/red raspberry juice. Yeast and molds cell structure adapted from Ref. [36] and PME reaction adapted from Ref. [59].

In this work, a putative effect of the HHP and PEF treatments on microbial safety, phytochemical parameters (pH, %AT, °Brix, and *a_w_* values), retention of vitamin C and phenolic compounds, and the enzyme inactivation of the PME activity of the black/red raspberry juice was proposed (Figure 2).

The microbial inactivation by HHP and PEF treatments was higher in yeast rather than molds. This was attributed to morphological change and electroporation by HHP and PEF, respectively. As well as the microorganism type and the HHP and PEF treatments. The effect on the pH, %AT, °Brix, phenolics, and vitamin C, suggest that HHP and PEF treatments improve the release of compounds from the cell organelles and compartments containing high concentrations of organic acids, vitamin C, or TPC. The increment in %TA and pH reduction is attributed to the organic acids released from the vegetable cell. In addition, the increment in °Brix is probably produced by sugar released to the polymer hydrolysis until its monomers. The physicochemical parameters’ results would be related to the microbiological analysis, due to the increment in TPC and vitamin C, which would also contribute to the inactivation of the microorganism as phenolics and vitamin C are compounds with antimicrobial activity [57,58].

Results also reveal that HHP and PEF treatments favor other biochemical reactions that lead to activation or inhibition of the PME activity (*p* < 0.05). The PME catalyzed the formation of insoluble calcium pectate gels which react with calcium ions (Ca^2+^) present in the juices, and then precipitate pulling the cloud with them causing juices clarification [60,61]. In this work, the gelation caused by Ca^2+^ (or other di/trivalent cations) cross-linking of demethylated pectin was activated by the HHP treatment at 400 MPa but inhibited by the HHP-treatments at 500–600 MPa, and the PEF treatments at 500 Hz/113.8 kJ/L, 100 Hz/79.7 kJ/L, and 200 Hz/168.4 kJ/L. This suggests that the effect on PME activity depended on the pressure and electrochemical effect applied. Due to this, the HHP and PEF produce changes in the structure and conformation of enzymes, resulting in the modification of their enzyme catalytic activities [62]. The pressure and the electrochemical effect produce the dissociation of the non-covalent, ionic, hydrophobic, and hydrogen bonds to change mainly the secondary (*α*-helix, *β*-sheets, etc.), tertiary (spatial conformation), and quaternary (number and arrangement of protein subunits) structures of the protein [63]. Thus, the increment of the PME activity at 400 MPa would be attributed to a reversible unfolding of the protein, while the reduction of the PME activity at 500–600 MPa would be attributed to an irreversible unfolding [62]. This unfolding leads to conformational changes, which can modify functionality resulting in a decrease or increase in the enzyme catalytic activity as observed in this work [10,11]. The high PME activity in the HHP treatment at 400 MPa would be also related to the high vitamin C content. It has been reported that vitamin C acts as an enzyme protective agent in fruits [52]. However, in PEF treatments this protective effect was not observed. Suggesting that both technologies produce a differential molecular or structural effect on the enzymes and metabolites.

The inhibition of the PME activity in the PEF treatment at 500 Hz/113.8 kJ/L, 100 Hz/79.7 kJ/L, and 200 Hz/168.4 kJ/L, is attributed to the breaking of the existing covalent bonds and non-covalent interactions in enzymes, resulting in a new sort of interactions within the peptide chains, which result in the loss of activity [64]. Thus, the PME inactivation shows that any changes in the protein interactions bring a change in enzyme activity due to modifications in the structure of active sites or overall three-dimensional molecular conformations of the enzyme, i.e., the HHP and PEF can induce the association or dissociation of functional moieties in proteins, which in turn can lead to protein denaturation. However, considering that covalent bonds are negligibly compressible under pressure and are generally unaffected by the pressures used in food processing. This means that many components responsible for the sensory and nutritional quality of the black/red raspberry juice, such as flavor components and vitamins, are not destroyed by HHP [65], and probably by PEF treatments, making both of these technologies of interest to the fruits juice industry.

## 5. Conclusions

This study showed the effect of the HHP and PEF processes on black/red raspberry juice. Both HHP and PEF are two non-thermal technologies that can be applied for microorganism inactivation and PME activity on fruit juice. Especially all HHP-treatments achieved the highest (*p* < 0.05) *log*-reduction of molds (*log* 1.85 to 3.72) and yeast (*log* 3.19), in comparison with PEF treatments. Important differences regarding the quality values of the pH, %AT, and TSS (◦Brix), attributed to the compounds’ decompartmentalization and release by HHP and PEF treatments were found. An increment in the TPC and vitamin C in the HHP and PEF treated juice was also observed. Both phytochemical compounds are associated with a fresh flavor; therefore, HHP and PEF increased the quality of the black/red raspberry juice. The optimum condition for the highest increment in TPC (79%) and vitamin C (77%) was the PEF treatment with 200 Hz and 168.4 kJ/L. At this PEF condition, the inactivation of the PME activity of 19% and microbial inactivation of molds (2.12-*log* reduction) and yeast (3.19-*log* reduction) were obtained. Conversely, the optimum HHP condition for the highest increment in vitamin C was 400 MPA-CUT, while, for microbial safety, it was 400 MPA−2 min (3.72-*log* reduction of molds and 3.19-*log* reduction of yeast), and for PME activity inactivation (22% of inactivation) the optimum HHP-condition was 600/10 min. Overall, the effect on the black/red raspberry juice was dependent on the HHP and PEF treatment applied, as well as, on the microorganisms and phytochemical compounds. In addition, a putative effect of HHP and PEF treatments on microbial safety, enzyme inactivation, and phytochemicals retention was also disused in detail. Further studies with more treatment conditions should be applied to reach higher enzyme inactivation. 

## Figures and Tables

**Table 1:** Effect of HHP and PEF treatments on log reduction of molds and yeast of black/red raspberry juice.

High Hydrostatic Pressure (HHP)					
Conditions		Molds		Yeast	
Pressure (MPa)	Time (min)	CFU/mL	*Log* (N_t_/N_0_)	CFU/mL	*Log* (N_t_/N_0_)
Control		5.30 × 10^3^ ± 0.01 c	3.72 ± 0.01 c	1.55 × 10^3^ ± 0.12 b	3.19 ± 0.00 b
400	CUT	7.50 × 10^1^ ± 0.21 b	−1.85 ± 0.21 b	1.00 × 10^0^ ± 0.00 a	−3.19 ± 0.00 a
	2	1.00 × 10^0^ ± 0.00 a	−3.72 ± 0.00 a	1.00 × 10^0^ ± 0.00 a	−3.19 ± 0.00 a
	5	1.00 × 10^0^ ± 0.00 a	−3.72 ± 0.00 a	1.00 × 10^0^ ± 0.00 a	−3.19 ± 0.00 a
	10	1.00 × 10^0^ ± 0.00 a	−3.72 ± 0.00 a	1.00 × 10^0^ ± 0.00 a	−3.19 ± 0.00 a
500	CUT	1.00 × 10^0^ ± 0.00 a	−3.72 ± 0.00 a	1.00 × 10^0^ ± 0.00 a	−3.19 ± 0.00 a
	2	1.00 × 10^0^ ± 0.00 a	−3.72 ± 0.00 a	1.00 × 10^0^ ± 0.00 a	−3.19 ± 0.00 a
	5	1.00 × 10^0^ ± 0.00 a	−3.72 ± 0.00 a	1.00 × 10^0^ ± 0.00 a	−3.19 ± 0.00 a
	10	1.00 × 10^0^ ± 0.00 a	−3.72 ± 0.00 a	1.00 × 10^0^ ± 0.00 a	−3.19 ± 0.00 a
600	CUT	1.00 × 10^0^ ± 0.00 a	−3.72 ± 0.00 a	1.00 × 10^0^ ± 0.00 a	−3.19 ± 0.00 a
	2	1.00 × 10^0^ ± 0.00 a	−3.72 ± 0.00 a	1.00 × 10^0^ ± 0.00 a	−3.19 ± 0.00 a
	5	1.00 × 10^0^ ± 0.00 a	−3.72 ± 0.00 a	1.00 × 10^0^ ± 0.00 a	−3.19 ± 0.00 a
	10	1.00 × 10^0^ ± 0.00 a	−3.72 ± 0.00 a	1.00 × 10^0^ ± 0.00 a	−3.19 ± 0.00 a
**Pulsed Electric Field (PEF)**					
**Conditions**		**Molds**			**Yeast**
**Frequency** **(Hz)**	**Specific Energy** **(kJ/L)**	**CFU/mL**	***Log* (N_t_/N_0_)**	**CFU/mL**	***Log* (N_t_/N_0_)**
Control		5.30 × 10^3^ ± 0.01 e	3.72 ± 0.01 e	1.55 × 10^3^ ± 0.002 c	3.19 ± 0.00 c
100	19.7	3.45 × 10^3^ ± 0.03 e	−0.19 ± 0.03 e	1.25 × 10^3^ ± 0.07 c	−0.09 ± 0.007 c
	79.7	2.50 × 10^2^ ± 0.002 c	−1.33 ± 0.002 c	6.50 × 10^1^ ± 0.14 b	−1.38 ± 0.14 b
200	42.9	6.30 × 10^2^ ± 0.06 d	−0.92 ± 0.06 d	1.00 × 10^0^ ± 0.00 a	−3.19 ± 0.00 a
	168.4	4.00 × 10^1^ ± 0.16 a	−2.12 ± 0.16 a	1.00 × 10^0^ ± 0.00 a	−3.19 ± 0.00 a
500	113.8	1.00 × 10^2^ ± 0.12 b	−1.72 ± 0.12 b	1.00 × 10^0^ ± 0.00 a	−3.19 ± 0.00 a

Mean value ± standard deviations (*n* = 3). Different letters indicate significant differences (*p* < 0.05). *Log* = logarithm base 10; CFU/mL= Colony Forming Units per milliliter; N_0_= the initial number of cells; t = time; N_t_ = number of cells after time t.

**Table 2:** Physicochemical parameters of the black/red raspberry juice HHP and PEF-treated.

High Hydrostatic Pressure (HHP)					
Conditions		pH	Titratable Acidity (% Citric Acid)	Water Activity (*a_w_*)	Total Soluble Solids (°Brix)
Pressure (MPa)	Time (min)				
Control			1.00 ± 0.00 c	0.98 ± 0.00 b	7.5 ± 0.00 a
400	CUT	3.43 ± 0.01 g	0.80 ± 0.00 a	0.98 ± 0.00 a, b	8.0 ± 0.00 b
	2	3.41 ± 0.01 f, g	0.80 ± 0.00 a	0.97 ± 0.00 a, b	8.0 ± 0.00 b
	5	3.36 ± 0.01 c	0.80 ± 0.00 a	0.98 ± 0.00 a, b	8.0 ± 0.00 b
	10	3.41 ± 0.01 f	0.80 ± 0.00 a	0.98 ± 0.00 b	8.0 ± 0.00 b
500	CUT	3.45 ± 0.00 h	0.80 ± 0.00 a	0.97 ± 0.00 a, b	8.0 ± 0.00 b
	2	3.42 ± 0.00 f, g	0.80 ± 0.00 a	0.97 ± 0.00 a, b	8.0 ± 0.00 b
	5	3.41 ± 0.00 f	0.80 ± 0.00 a	0.98 ± 0.00 a, b	8.0 ± 0.00 b
	10	3.39 ± 0.00 e	0.80 ± 0.00 a	0.98 ± 0.00 a, b	8.0 ± 0.00 b
600	CUT	3.41 ± 0.00 f	0.80 ± 0.00 a	0.97 ± 0.00 a, b	8.0 ± 0.00 b
	2	3.39 ± 0.00 e	0.80 ± 0.00 a	0.97 ± 0.00 a, b	8.0 ± 0.00 b
	5	3.41 ± 0.00 f	0.80 ± 0.00 a	0.96 ± 0.00 a,	8.0 ± 0.00 b
	10	3.43 ± 0.00 g	0.80 ± 0.00 a	0.97 ± 0.00 a, b	8.0 ± 0.00 b
**Pulsed Electric Field (PEF)**					
**Conditions**		**pH**	**Titratable Acidity (% Citric Acid)**	**Water Activity (*a_w_*)**	**Total Soluble Solids (°Brix)**
**Frequency (Hz)**	**Specific Energy** **(kJ/L)**				
Control		3.31 ± 0.00 b	1.00 ± 0.00 c	0.97 ± 0.00 a, b	7.5 ± 0.00 a
100	19.7	3.38 ± 0.00 d, e	0.90 ± 0.00 b	0.98 ± 0.00 b	8.5 ± 0.00 c
	79.7	3.38 ± 0.00 d, e	0.90 ± 0.00 b	0.98 ± 0.00 b	8.5 ± 0.00 c
200	42.9	3.36 ± 0.00 c	0.90 ± 0.00 b	0.97 ± 0.00 a, b	8.5 ± 0.00 c
	168.4	3.37 ± 0.00 c, d	0.90 ± 0.00 b	0.98 ± 0.00 b	8.5 ± 0.00 c
500	113.8	3.38 ± 0.00 d, e	0.90 ± 0.00 b	0.98 ± 0.00 b	8.5 ± 0.00 c

Mean value ± standard deviations (*n* = 3). Different letters indicate significant differences (*p* < 0.05).

## Data Availability

Data is contained within the article.
